# Influence of the Chin-Down and Chin-Tuck Maneuver on the Swallowing Kinematics of Healthy Adults

**DOI:** 10.1007/s00455-014-9580-3

**Published:** 2014-10-31

**Authors:** Ja-Ho Leigh, Byung-Mo Oh, Han Gil Seo, Goo Joo Lee, Yusun Min, Keewon Kim, Jung Chan Lee, Tai Ryoon Han

**Affiliations:** 1 Department of Rehabilitation Medicine, Seoul National University College of Medicine, 101, Daehang-ro, Jongno-gu, Seoul, 110-744 Republic of Korea; 2 Department of Biomedical Engineering, Seoul National University College of Medicine, 101, Daehang-ro, Jongno-gu, Seoul, 110-744 Republic of Korea

**Keywords:** Deglutition, Deglutition disorders, Rehabilitation, Biomechanics

## Abstract

The purpose of the study was to investigate the influence of the chin-tuck maneuver on the movements of swallowing-related structures in healthy subjects and formulate standard instructions for the maneuver. A total of 40 healthy volunteers (20 men and 20 women) swallowed 10 mL of diluted barium solution in a “normal and comfortable” position (NEUT), a comfortable chin-down position (DOWN), and a strict chin-tuck position (TUCK). Resting state anatomy and kinematic changes were analyzed and compared between postures. Although angles of anterior cervical flexion were comparable between DOWN (46.65 ± 9.69 degrees) and TUCK (43.27 ± 12.20), the chin-to-spine distance was significantly shorter in TUCK than in other positions. Only TUCK showed a significantly shorter anteroposterior diameter of the laryngeal inlet (TUCK vs. NEUT, 14.0 ± 4.3 vs. 16.3 ± 5.0 mm) and the oropharynx (18.8 ± 3.1 vs. 20.5 ± 2.8 mm) at rest. The maximal horizontal displacement of the hyoid bone was significantly less in TUCK (9.6 ± 3.0 mm) than in NEUT (12.6 ± 2.6 mm; *p* < 0.01) or DOWN (12.1 ± 3.0 mm; *p* < 0.01). TUCK facilitated movement of the epiglottic base upward (TUCK vs. NEUT, 15.8 ± 4.7 vs. 13.3 ± 4.5 mm; *p* < 0.01). In contrast, DOWN increased the horizontal excursion of the epiglottic base and reduced movement of the vocal cords. These results quantitatively elucidated the biomechanical influences of the chin-tuck maneuver including reduced horizontal movement of the hyoid bone, facilitation of vertical movement of the epiglottic base, and narrowing of the airway entrance. Comparing DOWN and TUCK, only TUCK induced significant changes in the airway entrance, hyoid movement, and epiglottic base retraction.

## Introduction

Various neurological disorders and mechanical injuries can cause swallowing difficulties, namely dysphagia [1]. To alleviate dysphagia, restorative or compensatory approaches have been adopted [2]. Compensatory approaches include food modification, postural changes, and compensatory maneuvers. Postural changes and maneuvers are simple and effective ways of improving the safety and efficacy of swallowing in many cases. The chin-down, head rotation, head tilting, supraglottic swallowing, and Mendelsohn’s maneuvers are examples of commonly used strategies [2, 3].

Among these, the “chin-down” or “chin-tuck” posture has been recommended to various patients [4] with the expectation that it can reduce the risk of laryngeal penetration [5] or aspiration [6]. With the chin tucked, the anterior pharyngeal wall is pushed backward, thus narrowing the airway entrance. The vallecular space is also widened [1], which mitigates the risk of subglottic aspiration from premature food spillage. Welch et al. [7] reported that airway protection improves with the chin tucked by narrowing the laryngeal entrance. Bülow et al. [8] revealed that the chin-tuck posture decreased resting state distances from the hyoid bone to the larynx and mandible. Despite the wealth of studies, changes in the movement of swallowing structures in different neck postures have not been evaluated extensively. Considering that a specific neck posture can affect not only the anatomy at rest, but also dynamic physiology during swallowing, the current understanding of the chin-down effect does not provide a complete picture.

There have also been discrepancies in the terminology and practical instructions regarding the maneuver. Because there is no consensus on what an “effective” chin-down posture is, variations of its effects in the literature are inevitable [9]. Although patients are usually taught to “position the chin toward the chest and look down toward the knees,” instructions vary from clinic to clinic [1]. Understanding the exact biomechanical consequences of a specific posture can provide valuable clues for the development of standard instructions for the position.

This study aimed to investigate the influence of different chin-down postures on swallowing kinematics and to provide a more concrete rationale for the use of these maneuvers.

## Materials and Methods

### Subjects

The study group consisted of 40 healthy volunteers (20 men and 20 women) ranging in age from 26 to 79 years (mean ± SD, 52.9 ± 17.9 years). We stratified participants into three recruitment groups (10 subjects for those of 20–39 years old, 10 for those of 40–59 years old, and 20 for those older than 60 years old). Also, the same number of men and women were recruited in each group (i.e., 5 men and 5 women each). They had no symptoms or signs of swallowing problems and reported no history of neurologic disease such as cerebral infarction, syncope, or transient ischemic attack, or history of pulmonary disease. Each participant additionally filled out questionnaires about their previous medical history. The number of subjects needed ranged from 25 to 37 for all outcome measures to achieve a statistical power of ≥0.80 with an alpha level of *p* ≤ 0.05 based on power analyses. The study protocol was approved by the Institutional Review Board of our hospital, and all participants were informed of the potential experimental risks and signed an informed consent document before the study.

### Positions

**Fig. 1:**
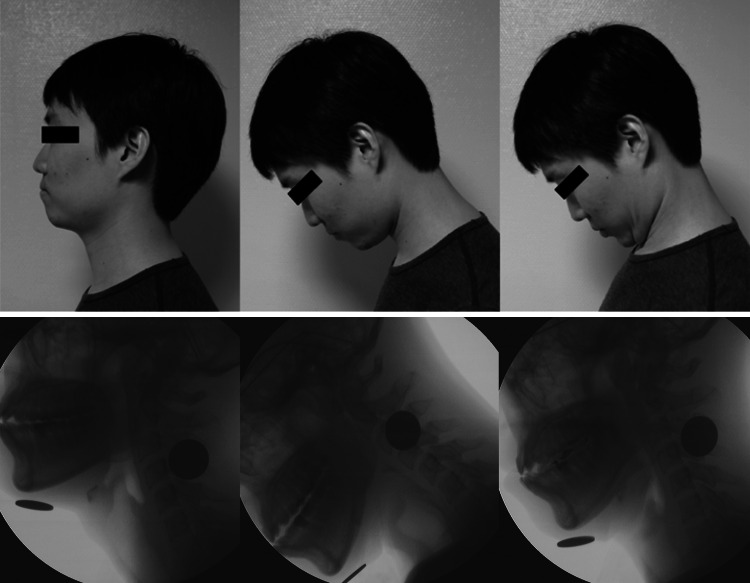
The instructions for the study postures (*upper pane*). The radiographic difference with each posture (*lower pane*)

Subjects were first instructed to drink the liquid in a “neutral” (NEUT) or “normal and comfortable” position. The instruction for the “comfortable chin-down” (DOWN) posture was “move your chin down” and for the “strict chin-tuck” (TUCK) posture, “tuck your chin as close to your sternum as possible” or “intentionally bring or touch your chin to your chest”, which was adapted from the instruction by Logemann [1]. The instructions were provided with brief illustrations (Fig. 1). No further explanation was given about the proposed mechanisms of the three different neck postures. Each subject was instructed in the positions and allowed to practice the swallow in those positions prior to imaging. The video fluoroscopic recording was performed in the order of NEUT, DOWN, and then TUCK. Only a single swallow in each position was recorded and analyzed from each subject.

### Videofluoroscopic Study (VFS) of Swallowing

**Fig. 2:**
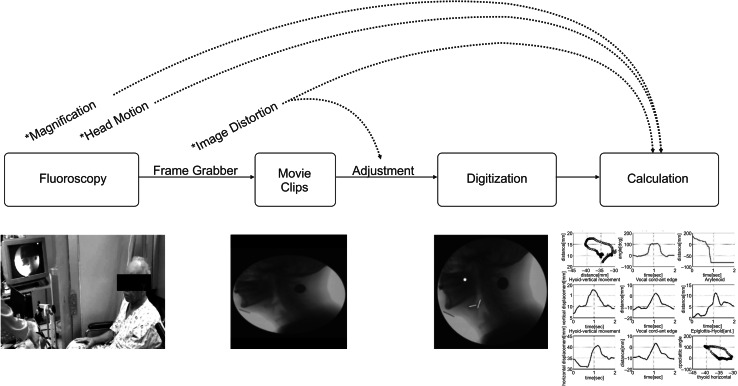
The process and potential errors of the kinematic swallowing analysis. An *asterisk* represents the potential error in each step, and a *dotted line* represents a correction of the error

The entire process of analysis is shown in Fig. 2. Images were acquired on a mobile fluoroscopy system (Medix 3000, Hitachi, Japan), and two-dimensional (2D) digitization of the swallowing motion was performed with the same system, as described previously [10]. Subjects were seated upright in a chair for the duration of the study and ingested 10 mL of 35 % w/v diluted barium solution (Solutop Suspension^®^, Tae Joon Pharm Corp., Ltd., Seoul, Korea) using a spoon. A coin 24 mm in diameter was taped under the subject’s chin at the midline to serve as a reference ruler for radiographic magnifications. All video clips were cropped from when the head of liquid reached the lower mandibular margin through the end of the liquid’s passage through the upper esophageal sphincter (UES).

**Fig. 3:**
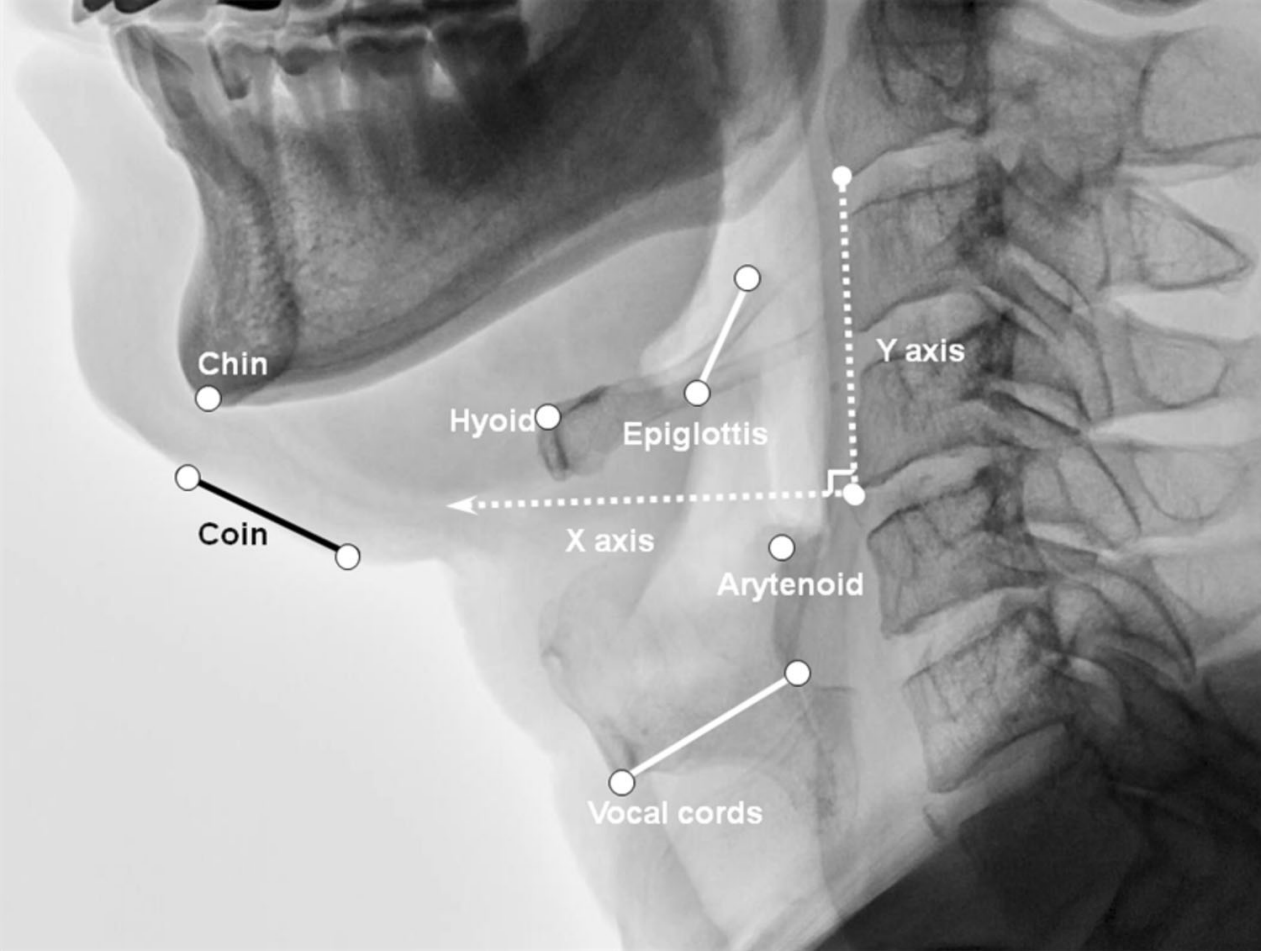
The coordination of the kinematic analysis and the selected anatomical points

A rater with 2 years’ research experience in swallowing motion analysis, who was blinded to the study design and purpose, analyzed the video clips. The following points of interest in each video frame were analyzed using motion analysis software (Ariel Performance Analysis System; Ariel Dynamics, Inc., Trabuco Canyon, CA, USA). The anterior–superior margin of the hyoid bone; base-to-tip of the epiglottis; head of the barium fluid; anterior–posterior margin of the mandible; anterior–posterior ends of the upper margin of the subglottic airway column, which represents the vocal cords; and the tip of the arytenoids were digitally coordinated in each frame, as was the mental protuberance in the resting position frame. To calculate the coordinates for each point, we operationally defined the y-axis as a straight line connecting the anterior–inferior border of the fourth cervical vertebra (the origin) to the anterior–inferior border of the second cervical vertebra; the x-axis was a straight line perpendicular to the y-axis crossing the origin (Fig. 3), as described previously [11, 12]. All the numbers, i.e., distances and excursions, were calculated and presented as the actual distances in millimeter. All digitized data were then filtered using a quintic spline algorithm. All binary data were exported for subsequent analysis. A script was written using MATLAB (R2007a, The MathWorks, Inc., Natick, MA, USA) for the adjustment of potential errors and calculations.

### Measurement

The following variables were measured: (a) the chin-cervical spine, epiglottic base-cervical spine, and epiglottic base-arytenoid distances (mm) at rest just before swallowing; (b) the maximal vertical and horizontal excursions (mm) of the hyoid, epiglottic base, and vocal cords (upper margin of the subglottic airway column), defined as the maximal distance from the starting point of each structure to the point of maximal excursion along each direction; the maximal 2D excursion of the hyoid, epiglottic base, and vocal cords, defined as the maximal distance from the starting point of each structure to the point of maximal excursion; and the maximal flip angle (degrees, °) of the epiglottis during swallowing; (c) the maximal vertical and horizontal 2D velocities (mm/s) of the hyoid, epiglottic base, and vocal cords, defined as the points with maximal velocity along each direction; and the velocity of the bolus head.

The distance from the “zero” point to the anterior–inferior border of the second cervical vertebra was calculated to serve as a covariate of the vertical spatial relation, and the distance from the mental protuberance perpendicular to the y-axis in the resting position as that of the horizontal spatial relation in the pharynx.

### Pooled Averages for Trajectories of the Hyoid Bone and Epiglottic Base

Pooled averages were calculated for the purpose of trajectory figure generation for the hyoid bone and epiglottic base. Because each subject spent different amounts of time performing the swallowing test, a temporal normalization was required. The digitized data from each subject were interpolated for a total of 100 time steps. In addition, to correct for anatomical differences among the subjects [13], a spatial normalization was implemented. The vertical length from C4 to C2 was normalized to 40 mm, and the horizontal distance from the chin to the cervical spine was normalized to 100 mm. After the temporal–spatial normalization, the pooled average trajectory and distributed range of motion were presented, respectively, as a series of mean values and band ranges obtained from elliptical distributions within 95 % confidence intervals (mean ± 2SEM) in both the x-axis and y-axis directions at each time step.

### Evaluation of Reliability

To evaluate the intra-rater reliability of the swallowing kinematics analysis, 10 VFS cases were utilized. Displacement, angle, and velocity for the hyoid, epiglottis, and vocal cords were calculated and compared. To determine intra-rater reliability, each rater analyzed the same cases twice at an interval of 1 month. Although there was an interval of just 1 month, the brightness characteristics of the images were slightly modified and the case names were changed prior to the second analysis.

### Statistical Analysis

A repeated-measure analysis of variance (RM-ANOVA) was used to compare variables among the posture subgroups using the within-subject effect. Variability in the size of body structures might have influenced the results, but did not reject the sphericity assumption of all variables. When there were significant differences among the groups, a Bonferroni-corrected pairwise comparison was used for the post hoc analysis according to the homogeneity of the variables. Values of *p* ≤ 0.05 were considered statistically significant for all comparisons. To evaluate intra-rater reliability, an intraclass correlation coefficient (ICC) was used. Statistical analyses were performed with SPSS 17.0 for Windows (SPSS Inc., Chicago, IL, USA). Values are given as the mean (standard deviation).

## Results

Distances from the chin to the posterior pharyngeal wall were measured to confirm the differences among the three postures, and were significantly different between TUCK and NEUT (*p* < 0.001) and between TUCK and DOWN (*p* < 0.001) postures.

### The Hyoid Bone

**Fig. 4:**
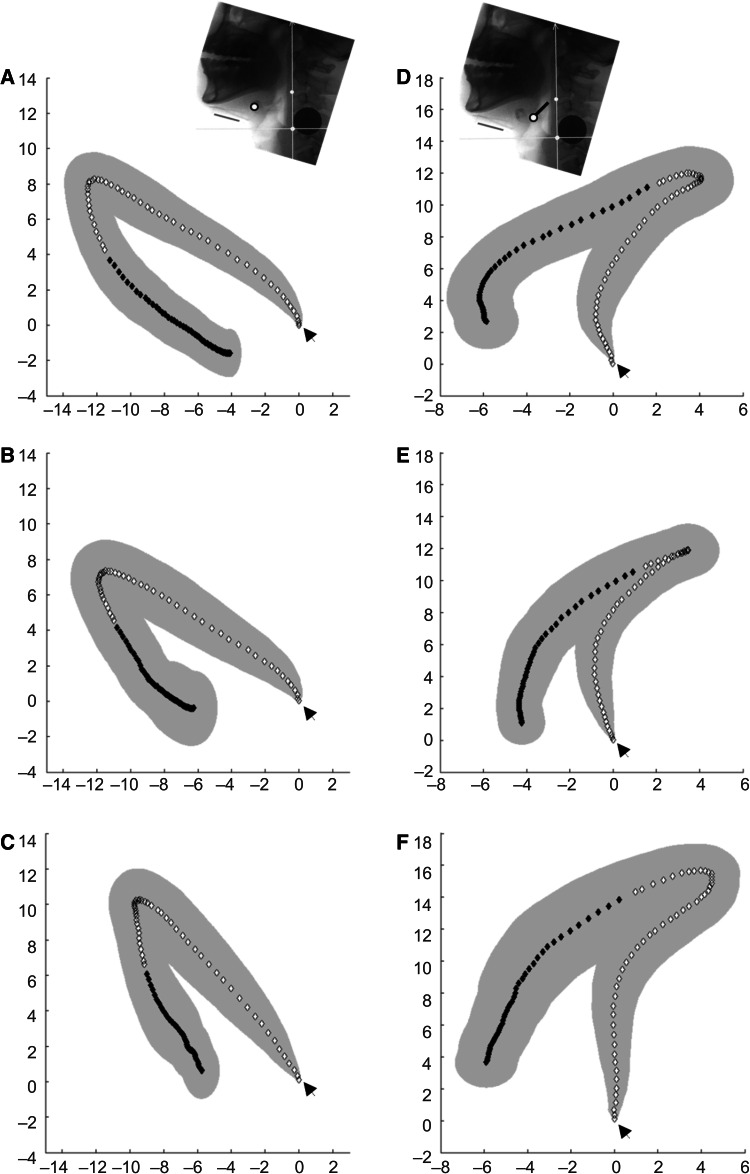
The pooled average trajectories of the hyoid bone and epiglottic base *left column*. *Arrow heads* in each trajectory indicate the starting point. The hyoid movement trajectories during swallowing in a neutral (**a**), a comfortable chin-down (**b**), and a strict chin-tuck (**c**) posture are shown. The chin-tuck posture shows a marked reduction in hyoid excursion in the *horizontal* *direction* and a slight increase in the *vertical* *direction* (**c**). *Right column*. The trajectories of the epiglottic base during swallowing in a neutral (**d**), a comfortable chin-down (**e**), and a strict chin-tuck (**f**) posture are shown. The backward retraction and elevation of the epiglottic base is distinctively enhanced (**f**)

Mean displacement magnitudes and standard deviations of the hyoid bone and larynx in NEUT were similar to data reported in previous kinematic analysis studies [14]. Table 1 shows the changes in spatial variables for each posture. The distance of maximal horizontal excursion of the hyoid bone was significantly less in TUCK than in NEUT (*p* < 0.001) or DOWN (*p* < 0.001) postures. However, the maximal vertical displacement of the hyoid bone was not significantly different among the three postures. The maximal 2D excursion distance of the hyoid bone exhibited borderline significance between NEUT and TUCK (*p* = 0.059) positions. Figure 4 shows an example of the change in trajectory of the hyoid bone in each posture.

**Table 1:** Spatial variables for neutral, comfortable chin-down, and chin-tuck postures

	Neutral	Comfortable chin-down	Strict chin-tuck
Distance at rest (mm)			
Epiglottic base-posterior wall^*^	19.70 (3.28)^e^	19.38 (4.25)^f^	17.89 (3.26)^e,f^
Epiglottic base-arytenoid^*^	16.36 (5.00)^e^	15.72 (4.90)	14.03 (4.27)^e^
Hyoid bone-vocal cord^*^	36.92 (6.19)^d,e^	34.14 (6.73)^d,f^	30.78 (7.22)^e,f^
Chin-posterior wall^*^	80.66 (7.00)^e^	80.19 (8.13)^f^	74.67 (8.22)^e,f^
Hyoid bone, maximal excursion (mm)			
Vertical	11.43 (4.80)	10.63 (5.21)	12.02 (4.89)
Horizontal^*^	12.55 (2.59)^e^	12.14 (2.99)^f^	9.64 (3.03)^e,f^
2D	14.59 (3.43)	14.29 (3.89)	13.93 (3.92)
Epiglottic base, maximal excursion (mm)			
Vertical^*^	13.33 (4.48)^e^	13.43 (4.68)^f^	15.79 (4.67)^e,f^
Horizontal^*^	10.73 (3.97)^d^	12.66 (4.52)^d^	11.61 (3.72)
2D (Posterior-upward)^*^	13.26 (4.71)^e^	14.16 (4.91)^f^	16.25 (4.38)^e,f^
Epiglottic flip angle, degrees (°)	111.37 (21.65)	118.55 (22.32)	119.68 (31.31)
Vocal cords, maximal excursion (mm)			
Vertical^*^	21.13 (5.56)^a^	18.86 (5.82)^a^	19.01 (6.18)
Horizontal^*^	6.64 (2.13)^b,d^	4.69 (1.55)^d^	5.32 (2.61)^b^
2D	21.84 (6.28)^a,b^	19.50 (5.65)^a^	19.24 (6.83)^b^

*2D* two-dimensional

* *p* < 0.01 using a repeated-measure ANOVA

^a,b,c^Significantly different at *p* < 0.05 by Bonferroni-corrected pairwise comparisons

^d,e,f^Significantly different at *p* < 0.01 by Bonferroni-corrected pairwise comparisons

### The Epiglottis and the Laryngeal Entrance

The distance between the epiglottic base and the arytenoids in the lateral fluoroscopic projection, which represents the anteroposterior diameter of the laryngeal entrance, was narrower in TUCK than in NEUT (*p* = 0.001) or DOWN (*p* = 0.001) postures. The minimum distance from the epiglottic base to the posterior pharyngeal wall was narrower in TUCK than in NEUT (*p* < 0.001) or DOWN (*p* < 0.001) postures. TUCK showed greater vertical and 2D excursions of the epiglottic base compared to NEUT (*p* = 0.004 and *p* < 0.001) and DOWN (*p* = 0.006 and *p* < 0.001) postures. In contrast, DOWN, not TUCK, showed a greater horizontal excursion of the epiglottic base than NEUT (*p* = 0.025). The maximal angle of epiglottic rotation also increased from NEUT to DOWN and TUCK, but the trend was not significant.

### The Vocal Cords

The displacement of the upper margin of the subglottic airway column, which represents the vocal cords’ motion during swallowing, was reduced in both DOWN and TUCK postures. As compared to NEUT posture, DOWN resulted in a reduction of the vertical (*p* = 0.026) and horizontal (*p* < 0.001) displacements of the vocal cords. TUCK showed reduced horizontal displacement of the vocal cords (*p* = 0.049).

### Movement Velocities of Anatomical Structures During Swallowing

Table 2 shows the velocities of the swallowing structures. TUCK resulted in a reduced maximal horizontal velocity of the hyoid bone compared to NEUT (*p* = 0.001) and DOWN (*p* = 0.025) postures. During swallowing, the epiglottic base moves upward and backward, to the nasopharynx and posterior pharyngeal wall, respectively. Swallowing in TUCK generated a faster upward velocity of the epiglottic base (*p* = 0.016).

**Table 2:** Maximal velocities of the swallowing structures

	Neutral	Comfortable chin-down	Strict chin-tuck
Hyoid, maximal velocity (mm/s)			
Horizontal^*^	55.77 (30.45)^b^	50.36 (29.90)^c^	36.71 (20.37)^b,c^
Vertical	53.26 (22.88)	56.11 (25.47)	60.83 (35.74)
2D	125.16 (51.12)	113.53 (43.18)	104.51 (57.06)
Epiglottic base, maximal velocity (mm/s)			
Horizontal	83.88 (49.31)	83.44 (45.44)	77.73 (35.07)
Vertical	72.82 (32.28)^a^	86.86 (41.47)	98.68 (49.02)^a^
2D	127.76 (54.28)^a^	148.95 (72.51)	159.16 (73.63)^a^
Vocal cords, maximal velocity (mm/s)			
Horizontal	28.67 (16.48)	32.72 (13.53)	35.29 (25.27)
Vertical	103.76 (44.80)	90.91 (53.38)	91.14 (49.16)
2D	109.10 (42.57)	101.19 (52.08)	105.45 (60.56)

*2D* two-dimensional

* *p* < 0.01 using a repeated-measure ANOVA

^a^Significantly different at *p* < 0.05 by Bonferroni-corrected pairwise comparisons

^b,c^Significantly different at *p* < 0.01 by Bonferroni-corrected pairwise comparisons

### Intra- and Inter-Rater Reliability

In the determinations of the two raters, all measurements, except for the maximal horizontal velocity of the hyoid bone (0.717, 0.792), displayed almost perfect intra-rater reliability coefficients that ranged from 0.894 to 0.997. Inter-rater tests of the measurements also showed comparable reliabilities from 0.748 to 0.995.

## Discussion

In the present study, we demonstrated that the movements of the pharyngeal and laryngeal structures during swallowing are differentially influenced by head and neck postures. We evaluated not only the maximal excursion distances and velocities, but also the trajectories of the major structures. The in-depth analysis in this study revealed that the forward flexion of head and neck in the TUCK posture reduced the anteroposterior distance of the oropharynx as well as the laryngeal inlet at rest; whereas the DOWN posture had no effect on these distances. In terms of dynamic motions during swallowing, TUCK restricts the maximal horizontal excursion of the hyoid bone, epiglottic base, and larynx. The peak velocity of the horizontal excursion of the hyoid bone was also reduced in the TUCK posture. The maximal vertical and 2D displacements of the epiglottic base were significantly increased in the TUCK posture. On the other hand, DOWN restricted the vertical and horizontal excursion of the larynx compared to the NEUT posture. In addition, the horizontal excursion of the epiglottic base was increased in the DOWN posture.

The most unique feature of the present study was that our method presented the locations of the major anatomical structures at each time point during swallowing in different head and neck postures. In this way, our study demonstrated the trajectories of the structures as well as vertical and horizontal components of the movements, which have not been previously reported with regard to these postures.

The epiglottic movements during swallowing, including the tilt angle and base movement, were also novel findings.

Traditionally, the “chin-down” or “chin-tuck” posture has been known to reduce the risk of aspiration by narrowing the airway entrance [7]. The distance from the epiglottic base to the arytenoid, which represents the laryngeal inlet, is one of the most important markers for airway protection [15, 16]. The present study showed that only TUCK, not DOWN, reduced the laryngeal inlet at rest. In addition, our results suggest that TUCK may ease swallowing in patients with weak tongue-base retraction by reducing the width of the oropharynx.

The TUCK posture inhibited horizontal hyoid bone movement. This can be explained by the tongue and submental muscles being compressed by the mandible in the TUCK posture. Another explanation is that a reduced excursion distance can result from decreased resting muscle length. Muscle operates with greatest contractile force when close to its resting length in an anatomical position [17]. In TUCK, the submental muscle length is shorter than in NEUT. The reason why the maximal horizontal velocity of the hyoid bone was reduced significantly may be understood in this way. Therefore, it should be noted that TUCK may deteriorate the UES opening because horizontal hyoid motion plays an important role in the opening of the UES [18]. On the other hand, DOWN had no significant effect on hyoid bone movement, which suggested that this comfortable posture did not compress or shorten these muscles.

We suppose that the epiglottic base can, at least in part, play a role as a surrogate marker for tongue base movement, because it is located at the lower end of the tongue base. In NEUT, the epiglottic base initially moved upward and backward, then descended toward the anterior (Fig. 4d). The initial upward and backward movement may represent tongue base retraction in the pharyngeal phase of swallowing. Our results suggest that TUCK enhances tongue base retraction, while DOWN does not, because the 2D excursion distance of TUCK was greater than in NEUT or DOWN, and the 2D velocity of TUCK was greater than that of NEUT (Table 1, Figs. 4e, f). According to a previous study concerning the mechanism of epiglottic tilt, a superior movement of the thyroid cartilage compresses the pre-epiglottic fat pad, which limits the downward movement of the epiglottic base during swallowing [19]. TUCK may enhance the dynamic compression of the fat pad, which leads to increases in the vertical and 2D movement of the epiglottic base. As the tongue base is known as a major pressure generator in swallowing [20], great and rapid tongue base retraction in TUCK can exert a higher pressure on the descending bolus.

The laryngeal motions were decreased in both TUCK and DOWN compared to NEUT, although it was more remarkable in DOWN. Reduced hyolaryngeal elevation is usually considered a negative finding that can cause impaired airway protection [21, 22]. However, Bülow et al. [8] reported that the chin-tuck posture effectively decreases the distance of the anatomical structures, which causes shortening of the route necessary for laryngeal elevation. Because the laryngeal inlet was shortened in TUCK, reduced laryngeal motion might be sufficient to protect the airway from aspiration. Therefore, TUCK may be helpful for dysphagic patients with decreased laryngeal motion.

In terms of pressure, previous studies that measured the pressure of pharyngeal constriction and pharyngoesophageal space by manometry give us clues to pressure changes in accordance with neck posture changes [8, 23–25]. Bülow et al. [19] reported that chin-tuck posture can increase inferior pharyngeal sphincter pressure in healthy subjects, but not in patients with pharyngeal dysfunction. Recently, McCulloch [23] and Balou [25] examined manometric studies on both chin-down and chin-tuck postures, which revealed that a more tucked posture increased the duration of relaxation and decreased UES pressure. When we refer to their findings in light of the present study, TUCK resulted in no changes in total excursion, but the influence of horizontal (anterior) movement waned, which resulted in decreased UES pressure. A previous study emphasized the role of horizontal (anterior) hyoid movement on the opening of the UES [18].

Considering that our study has broad age spectrum of subjects and swallowing physiology might differ along the age, we performed subgroup analysis in two age groups (patients aged <60 years, and aged ≥60 years). Younger age group showed more definite differences on the maximal excursion of three anatomical structures. The difference of hyoid horizontal excursion of three postures was similar with whole group analysis. Additionally, vertical displacement was markedly increased in TUCK than DOWN. The vertical and 2D excursion distance of TUCK of young age groups was also greater than in NEUT or DOWN, which is comparable with the results of whole group analysis. The laryngeal motions were smaller in DOWN as compared to NEUT, but not in TUCK. Older group showed similar trends with results on Table 1 and 2, but statistical significance was compromised on all variables about the epiglottis and vertical displacement of the vocal cords.

Our results, which showed kinematic differences between DOWN and TUCK postures, suggest that we should emphasize patient education on correct postures. Okada et al. [9] revealed that clinicians have various understandings of the same posture and that a single-term represented more than two postures. Furthermore, they questioned the effect of different head and neck positions with ambiguous terms, which resulted in unstandardized effects of swallowing. The results of the effectiveness of the chin-down and chin-tuck postures are controversial between studies. A recent study of 176 healthy volunteers that calculated the average angle of the chin-down posture reported that simple instructions could achieve an angle in the range reported by Welch et al. [7] as yielding a clinical benefit in radiographic studies [26]. On the other hand, subtle changes in neck flexion and head flexion produced quite different changes in the kinematics of the pharyngeal structures [9]. Comparisons among head flexion only, neck flexion only, and head and neck flexion could provide more information about the effects of various postures. Although we initially tried a head flexion only posture, there was no obvious chin position point, and individual variations in neck circumference and head flexibility also influenced the posture. A previous study that measured the craniovertebral angle formed by the MacGregor plane and the odontoid plane [27] (occiput-C2) reported no changes in the anteroposterior or vertical hyoid bone position from changes in head posture. Because this result demonstrated that head flexion only was not enough to create an effect, we hypothesized that the combination of both head and neck flexion could meaningfully affect the positions and movements of the laryngeal and pharyngeal structures. As a result, DOWN and TUCK postures combined both head and neck flexion.

## Limitations

A limited number of swallowing trials and individual variability remain methodological concerns. The present study analyzed only one swallow per posture. To reduce variability, volunteers in this study practiced a dry swallow three times in each posture and swallowed a barium bolus without radiation before capturing one swallow for image analysis. The mean values of each kinematic parameter may be influenced by one swallow per posture and the individual variability in a single volunteer.

The anatomical reference for the coordinate system also could be a limitation. The y-axis was defined as the line connecting the anterior–inferior corners of the C2 through C4 vertebrae. When the volunteer flexes their neck, the axis can change through bending of the vertebral alignment, which could lead to changes in vertical and horizontal coordinate values. Using C2-C4 as the y-axis is not a gold standard, but an optimal standard, because this segment is less influenced than the maxilla or the mandible by chin movement and is a more inert segment than other spinal segments including C4-C5 and C5-C6, where the greatest amount of neck flexion occurs [28, 29].

To measure the possible bias following flexion, each axis rotation angle (ARA) of the C2-C4 axis was analyzed against the true vertical axis in each posture. The mean ARA in NEUT had a significantly smaller angle (11.6 ± 6.5 degrees) than in TUCK (43.3 ± 12.2 degrees, *p* < 0.002) or DOWN (45.7 ± 9.7 degrees, *p* < 0.001) postures, but a significant difference was not observed between TUCK and DOWN.

## Conclusion

This study substantiates the alleged effects of the chin-tuck maneuver through quantitative kinematic data such as maximal displacements, velocities, and tilt angles, which verify the difference between the chin-tuck and similar chin-down postures. The chin-down posture has no remarkable effect, except on horizontal epiglottic movement. In contrast, the exact chin-tuck posture represents distinct kinematics from the neutral and chin-down postures, and facilitates airway protection and enhances tongue base retraction, but has the possibility of reducing the UES opening.

Therefore, in accordance with the patient’s pathologic severity, only the chin-down posture may be effective to adequately widen the vallecular space, and it is important to instruct patients in the exact chin-tuck posture, which can provide essential airway protection.
